# *Uncaria tomentosa* as a Promising Natural Source of Molecules with Multiple Activities: Review of Its Ethnomedicinal Uses, Phytochemistry and Pharmacology

**DOI:** 10.3390/ijms26146758

**Published:** 2025-07-15

**Authors:** Olinda Marques, Artur Figueirinha, Maria Eugénia Pina, Maria Teresa Batista

**Affiliations:** 1 University of Coimbra, Faculty of Pharmacy, 3000-548 Coimbra, Portugal; olindamarques@hotmail.com (O.M.); mtpmb@ff.uc.pt (M.T.B.); 2 Associated Laboratory for Green Chemistry (LAQV) of the Network of Chemistry and Technology (REQUIMTE), University of Coimbra, 3000-548 Coimbra, Portugal; 3 Chemical Engineering and Renewable Resources for Sustainability (CERES), Department of Chemical Engineering, University of Coimbra, 3030-790 Coimbra, Portugal; 4 Research Institute for Medicine (iMed.ULisboa), Faculty of Pharmacy, Universidade de Lisboa, 1649-004 Lisbon, Portugal

**Keywords:** *Uncaria tomentosa*, ethnobotany, alkaloids, phenolic compounds, triterpenes, phytochemistry, pharmacology

## Abstract

*Uncaria tomentosa* (*Ut*) is a Rubiaceae widely used in Peru’s traditional medicine. It is mainly known by the vernacular name of Cat’s claw due to its morphological aspects and is found in tropical low mountain forests of Central and South America. A decoction of *Ut* bark, root and leaves is used traditionally for different health problems, including arthritis, weakness, viral infections, skin disorders, abscesses, allergies, asthma, cancer, fevers, gastric ulcers, haemorrhages, inflammations, menstrual irregularity, rheumatism, urinary tract inflammation and wounds, among others, which gave rise to scientific and commercial interest. The present paper reviews research progress relating to the ethnobotany, phytochemistry and pharmacology of *Ut*, and some promising research routes are also discussed. We highlight the centrality of its different biological activities, such as antioxidant, anti-inflammatory, antiproliferative, antiviral, and antinociceptive, among others. Recently, studies of the health effects of this plant suggest that novel nutraceuticals can be obtained from it and applied as a preventive or prophylaxis strategy before the start of conventional drug therapy, especially for patients who are not prone to conventional pharmacological approaches to diseases. The present work emphasizes the current pharmacological properties of *Uncaria tomentosa*, evidencing its therapeutic benefits and encouraging further research on this medicinal plant.

## 1. Introduction

Humans have always sought and found remedies for illnesses in nature, usually in the plant kingdom. In fact, the use of medicinal plants in the prevention and treatment of diseases is as old as humanity. Indeed, archaeological evidence from the remains of our earliest ancestors indicates the use of various plants for medicinal purposes, dating back approximately 60,000 years. For millennia, the use of Phytotherapy corresponded to about 90% of the medicines used for the relief and cure of diseases [1].

Plants of the genus *Uncaria* have found widespread use in traditional medicine. *Uncaria* species are climbing vines of limited growth with claw-like thorns. There are at least 36 species referred to as Uncaria. *Uncaria tomentosa* (*Ut*) is a Rubiaceae used for at least 2000 years among some Peruvian tribes, especially the Asháninka [2].

Phytochemical studies have shown the presence of a wide range of bioactive compounds in various parts of *Ut* (bark, leaves, stem, and others), including alkaloids of the indole and oxindole type; triterpenes based on ursolic, oleanolic or quinovic acid; glycosides, sterols and phenolic compounds; phenolic acids; and flavan-3-ol related compounds like proanthocyanidins [3,4,5,6,7,8]. Currently, the pentacyclic and tetracyclic oxindole alkaloids (POA and TOA) are being used as chemical markers in quality control of raw material and derivatives from *Ut* [9], but other studies indicate that other phytochemicals contribute to its biological activities [10]. For instance, alkaloids isolated from the aqueous extracts of *Ut* roots were found to enhance the activity of phagocytes in vitro, and the results of another study suggested that some additional active phytochemicals may enhance immunity by selectively inducing apoptosis [11].

Nevertheless, significant activities have been exhibited by *Ut*, reinforcing the importance of this plant for the discovery of new drugs. *Ut* is consumed all over the world, mainly for its anti-inflammatory properties. So, its anti-inflammatory properties were verified at doses consistent with those used in traditional medicine, with different studies suggesting that they are the result of synergic activity [12,13]. Depending on the extraction procedure, the preparations of this plant often differ in their activity. An antiproliferative effect of *Ut* bark aqueous extracts was demonstrated for two human brain cell lines (neuroblastoma and malignant glioma) [14] and for human medullary thyroid carcinoma cells [15]. Antiviral activity was noted too; alkaloids, proanthocyanidins and quinovic acid glycoside are considered responsible for this activity [16]. A significant antimicrobial effect was also reported [17,18], but further phytochemical studies are required to determine the compounds that explain this effect. Aqueous extracts of bark from *Ut* seem to interact with distinct binding sites on the oestrogen receptor, which may be associated with its specific contraceptive effects, probably mediated by an interaction with 5-hydroxytryptamine 2 receptors (5-HT_2_) [14,19]. Its capacity of sunscreen protection was also demonstrated, actuating its ability to enhance natural repair [20].

The available toxicological studies suggest a low acute and subacute oral toxicity of aqueous extract from the root bark of *Ut* [21]. However, most studies have used *Ut* extracts without identifying the phytochemicals responsible for the activity or the existence of synergisms, which often occur in complex mixtures such as medicinal plant extracts.

For the present review, papers published until this date were collected from scientific electronic databases including Web of Science, Science Direct, and Google Scholar. Keywords such as: “*Uncaria tomentosa* or cat’s claw” and “traditional uses”, “phytochemicals”, “pharmacology”, “biological activities”, and “therapeutics” were used.

This scientific paper aimed to review the ethnobotany, phytochemistry and pharmacology aspects of *Ut* and provide the ongoing research, so important, on its pharmaceutical and medicinal applications, which would promote its future applications and achieve theoretical support for its further in-depth development.

## 2. Description and Ethnopharmacological Uses

*Uncaria tomentosa* can reach over 30 m in height [1] and has yellowish–white flowers [3]. The upper sides of the stipules in the buds are densely tomentose and the thorns are straight to sickle-shaped, very pungent and not sensitive [22]. This species grows at altitudes between 600 and 1000 m in tropical low mountain forests of Central and South America from Belize and Guatemala to Peru, Venezuela, Trinidad, Suriname [22], Colombia, Bolivia [1] and Brazil [3].

The most commonly known *Ut* name, cat’s claw, derives from its hook-like thorns that grow along the vine and resemble the claws of a cat [23]. The name cat’s claw refers to a multitude of species, which is reflected in the large number of drugs that are sold under this name in the street markets of Peru, all claiming to possess actions against a long list of diseases [24].

In Peru, a “*sancóshi*”—men, is prepared boiling approximately 20 g of sliced root bark of *Ut* in 1 L of water for 45 min. The liquid is decanted and losses are replenished. This bitter decoction is administered for 10 days [22]. *Ut* decoction is traditionally used for the treatment of asthma, rheumatism, arthritis, inflammation, gastritis, cancerous tumours, infectious diseases, abscesses, skin impurities, recovery from child birth, disease prevention and irregularities of the menstrual cycle [1,5,11,22,25,26]. Rumours of miraculous healings evoked scientific and commercial interest [27] and led to the development of over 50 *Ut* dietary supplements by 1997 in the United States [22].

Today, on the internet, it is easy to find this plant and to buy a great variety of commercial pills or capsules made of extracts or pulverised bark. Herbal drug preparations fall under the same official regulations as chemically defined substances concerning quality, efficacy, stability and safety [28]. However, in many herbal drug preparations, there is a lack of information about mechanisms of action, applications, appropriate doses, formulations [29] and also about stability, origin, part of the plant or even species used. That is what happens with *Ut* commercial preparations as well. Scientific evaluation and publication about the efficacy and toxicity of these natural products, especially human data, are still rare. Although *Ut* and its preparations are classified as nutritional and food supplements, it would be essential both scientifically and commercially to evaluate them both in animal and human studies [30]. This is especially concerning given that *Ut* is widely available online and it is indicated as a remedy for many different health problems, including arthritis, weakness, viral infections, skin impurities, contraception, abscesses, allergies, asthma, cancer, chemotherapy side effects, diseases prevention, fevers, gastric ulcers, haemorrhages, inflammations, menstrual irregularity, and recovery from child birth, rheumatism, urinary tract inflammation, and wounds, among others. The therapeutic actions already demonstrated will be described throughout this manuscript.

## 3. Phytochemistry

Phytochemical studies have shown the presence in *Ut* of a diverse range of bioactive metabolites, such as alkaloids, triterpenes, phenolic compounds and steroids.

### 3.1. Alkaloids

The alkaloids form a heterogeneous group of organic substances whose more significant molecular similarity is the presence of nitrogen, usually in the form of amine. There are several classes of alkaloids, and all have some physiological action in humans and in other animals, which has been used for the benefit of humans in the production of medical drugs.

**Figure 1:**
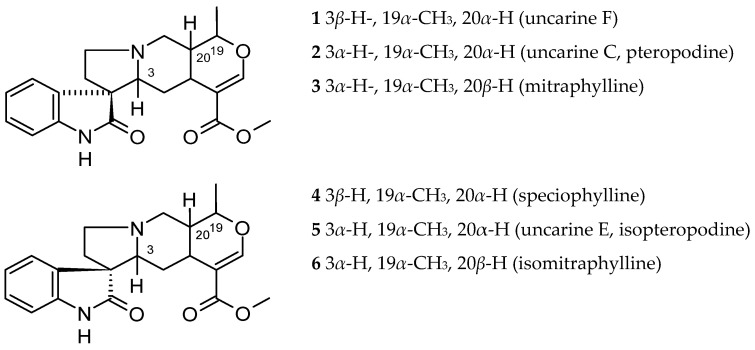
*Uncaria tomentosa* pentacyclic oxindole alkaloids (POA).

**Figure 2:**
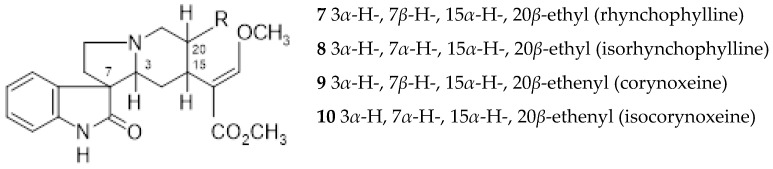
*Uncaria tomentosa* tetracyclic oxindole alkaloids (TOA).

**Figure 3:**
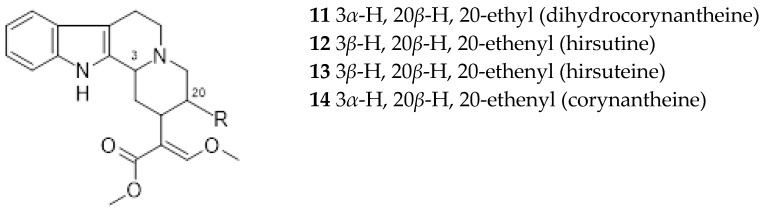
*Uncaria tomentosa* tetracyclic indole alkaloids.

**Figure 4:**
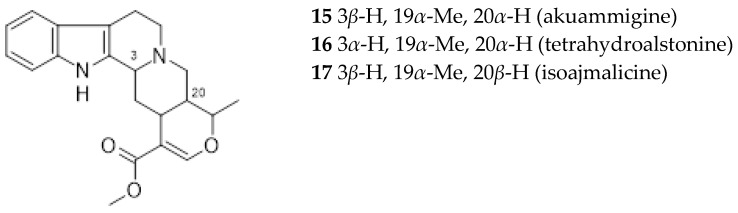
*Uncaria tomentosa* pentacyclic indole alkaloids (POA).

**Figure 5:**
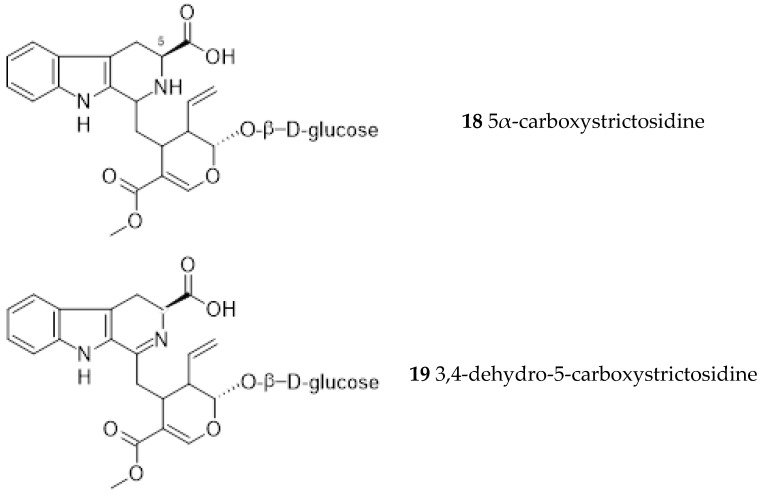

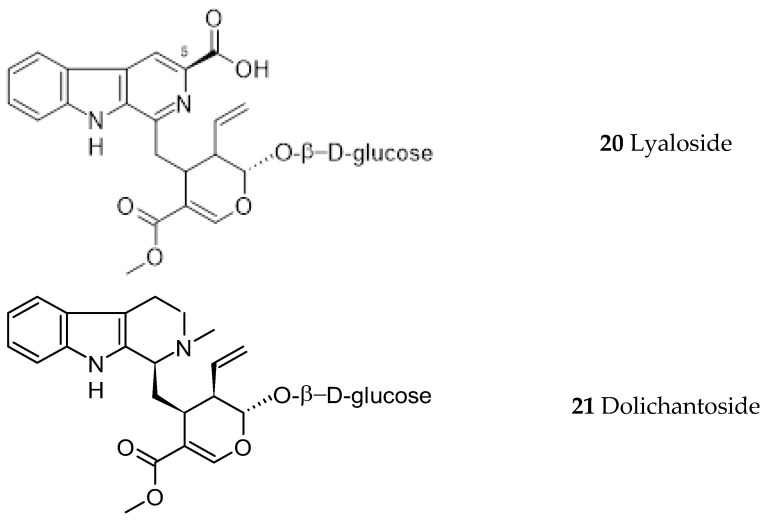
*Uncaria tomentosa* gluco-indole alkaloids.

In *Ut*, the majority of alkaloids are of the indole and oxindole type (Figure 1, Figure 2, Figure 3, Figure 4 and Figure 5). Attempts to extract potentially therapeutic phytochemicals from this plant led to the discovery of two chemotypes of *Ut*, with a different pattern of tetracyclic (TOA) and pentacyclic oxindole alkaloids (POA). Pentaciclic oxindole alkaloids are considered marker compounds of *Ut*. They are uncarine F (1), uncarine C (pteropodine) (2), mitraphylline (3), speciophylline (4), uncarine E or isopteropodine (5) and isomitraphylline (6) [3].

Rhyncophylline (7) and isorhyncophylline (8), along with their congeners corynoxeine (9) and isocorynoxeine (10), are tetracyclic oxindole alkaloids found in the TOA chemotype. In the leaves and roots of the tetracyclic indole alkaloids chemotype were also found small amounts of dihydrocorynantheine (11) and hirsutine (12), traces of hirsuteine (13), and exceptionally corynantheine (14). It was assumed that the oxindole alkaloids were synthesized from the steriochemically corresponding indole alkaloid akuammigine (15). *Ut* was also found to contain other alkaloids in small amounts, such as tetrahydroalstonine (16) and isoajmalicine (17) [31].

The tetracyclic and pentacyclic indole and oxindole alkaloids distribution in various parts of this plant was verified and is summarized in Table 1. In the young leaves were observed the highest concentrations of uncarine F (1) and lower doses of pteropodine (uncarine C) (2), speciophylline (4) and isopteropodine (uncarine E) (5), while in mature leaves, speciophylline was more abundant [31]. Mitraphylline (3) occurred in some of the older leaves, and isomitraphylline (6) in the stem bark [32].

Further studies led to the isolation of 5α-carboxystrictosidine (18) from the root bark [8]. A new gluco-indole alkaloid, 3,4-dehydro-5-carboxystrictosidine (19), was obtained from the stem of *Ut* together with 5α-carboxystrictosidine (18) and lyaloside (20) [33].

Later, dolichantoside (21) (not previously found in *Ut*) was detected in micropropagated plantlet roots, isolated and characterized from root suspension cultures [34].

### 3.2. Phenolic Compounds

**Figure 6:**
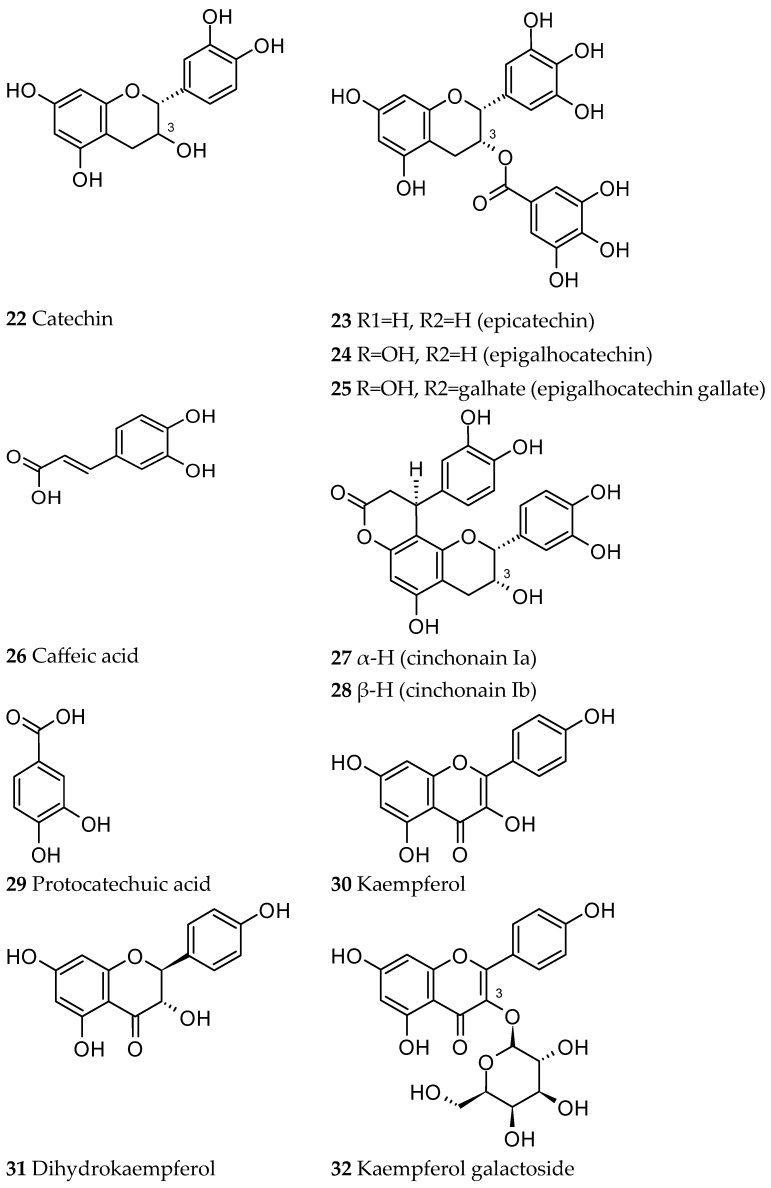
Phenolic compounds identified in *Ut*.

Condensed tannins are dimeric or polymeric macromolecules. Their basic structural element is a flavan-3-ol, like catechin (22), epicatechin (23) and epigallocatechin (24), which were isolated from the *Ut* bark, as well as the epigallocatechin gallate (25) and the flavonolignans cinchonain Ia and Ib (27 and 28) (Figure 6) [35,36].

An HPLC profile of an *Ut* bark decoction illustrated that it is mainly composed of phenolic acids, with caffeic acid (26) being the most representative, but also protocatechuic acid (29), catechin (22), epicatechin (23) and other flavan-3-ol related compounds, like oligomers (dimers and trimers) and more polymerised proanthocyanidins (Figure 6) [4].

Other works have shown the presence of flavonoids like kaempferol (30), dihydrokaempferol (31) [37], and trifolin (kaempferol galactoside) (Figure 6) (32) [38].

### 3.3. Terpenoids

**Figure 7:**
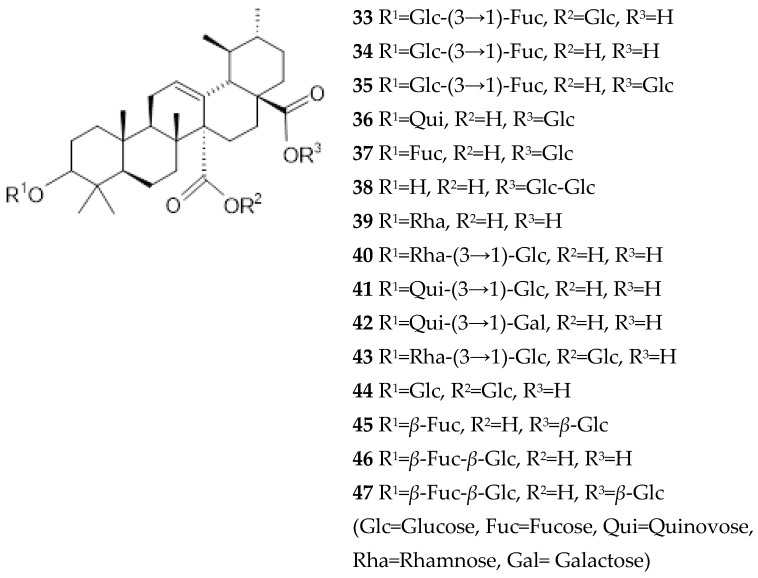
Quinovic acid glycosides isolated from *Uncaria tomentosa*.

**Figure 8:**
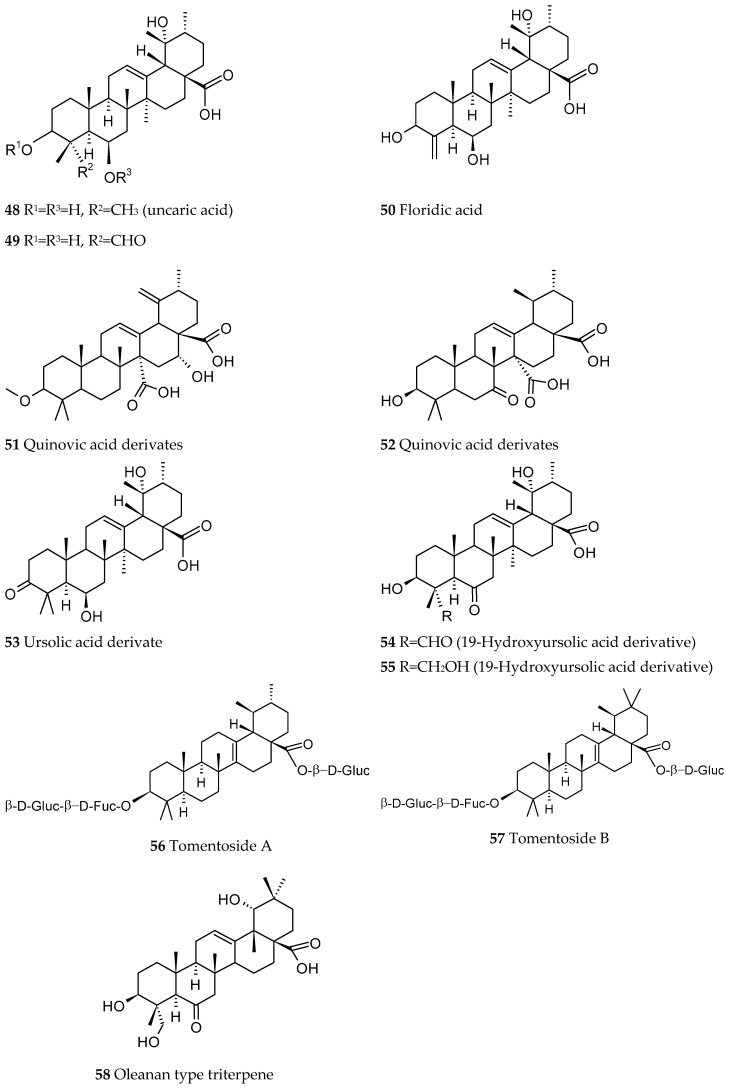
Triterpenes isolated from *Uncaria tomentosa*.

Terpenoids, also known as terpenes, and their related compounds are natural products with carbon skeletons formally derived from isoprene. They contain oxygen in various functional groups, double bonds, and generally one or more rings. This class is subdivided into hemiterpenes (the smallest of all with one unit of isoprene), monoterpenes, sesquiterpenes, diterpenes, sesterterpenes, triterpenes, tetraterpenes, and polyterpenes. In *Ut*, terpenoids are mainly triterpenes based on ursolic, oleanolic or quinovic acid structures, in which some glycosylation can be found (Figure 7 and Figure 8).

Fifteen quinovic acid glycosides (Figure 7) and three polyhydroxylated triterpenes, uncaric acid (48), 3*β*-, 6*β*-, 19*α*-trihydroxy-23-oxo-urs-12-en-28-oic acid (49) and floridic acid (50), were identified in the bark of *Ut* [5,39]. Three new polyoxigenated triterpenes based on ursolic or quinovic acid structures were isolated (51–53) [5].

Two new triterpenes with a 19-hydroxyursolic acid skeleton were also identified (54, 55) [40] along with two new nor-triterpene-glycosides derived from pyroquinovic acid, tomentosides A (56) and B 57) [41], a new oleanan type triterpene (58) and three new cincholic acid glycosides [42]. In the bark, an iridoid glycoside 7-deoxyloganic acid [43] and a triterpene, 7α-acetoxydihydronomilin [38], were identified.

### 3.4. Steroids

**Figure 9:**
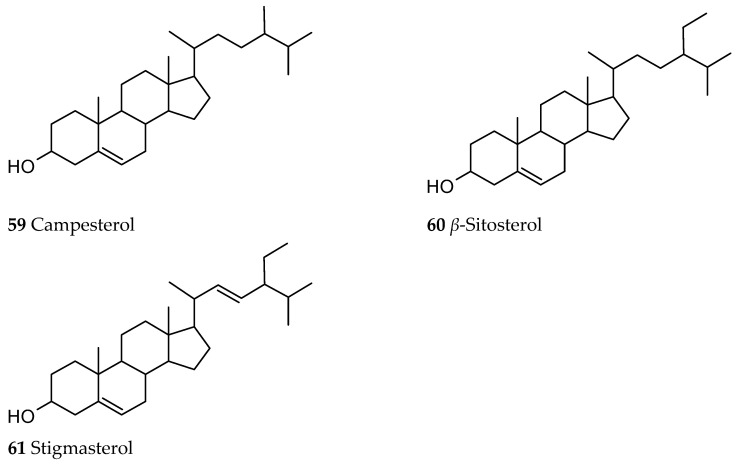
Sterols isolated from *Ut*.

A steroid fraction of *Ut* was also investigated showing the presence of campesterol (59), *β*-sitosterol (60) and stigmasterol (61), with *β*-sitosterol being the main sterol (Figure 9) [44].

## 4. Pharmacological Activity

This section summarizes the pharmacological activities and molecular mechanisms of *Ut* phytochemicals.

The majority of biological activities observed with extracts of *Uncaria tomentosa* have been attributed to alkaloids. Most commercial preparations are based on the oxindole alkaloid content [22] and many pharmaceutical firms standardize their *Ut* preparations for high dominance of pentacyclic alkaloids. Also, Ashaninka healers who have been familiar with *Ut* for over two thousand years use only the plants that represent the pentacyclic chemotype [22] and call them savéntaro (saveshi = plant, anteáro = potent) [24]. In fact, pentaciclic oxindole alkaloids have been referred to as exhibiting several activities, such as immunomodulatory, cytotoxic, anti-AIDS and antiamyloidose (Alzheimer’s disease) [43].

However, there are some important studies about the health benefits of other phytochemicals in *Ut*. In fact, more recently, water soluble *Ut* bark extracts, prepared in a manner similar to South America Native use, in which the plant parts are boiled in water overnight before being administered orally [20], and without significant amounts of alkaloids (<0.05%) still prove to be very effective for the treatment of several health disorders, including chronic inflammation and gastrointestinal dysfunction [45].

Furthermore, in a study using C-Med-100™, a water soluble extract from *Ut* formulated to mimic historical medicinal use was found to contain quinic acid and quinic acid esters (QAEs) [45], which are water soluble and as such are directly available for absorption. However, it was verified that relatively high concentrations of quinic acid are required to obtain a similar in vivo effect as with C-MED-100™ itself [46], suggesting that other phytochemicals can contribute to the biological response via synergism with quinic acid. In fact, some authors believe that diverse activities of *Ut* depend on a synergic effect among its different bioactive compounds [47].

Several pharmacological activities of *Ut* will be discussed in the following sections.

### 4.1. Antioxidant Activity

Antioxidants play a crucial role in the pathogenesis and prevention of numerous diseases [35], as they can mitigate oxidative damage caused by reactive oxygen species (ROS) to essential biomolecules such as DNA, lipids and proteins. Continuous exposure to a wide variety of chemically and structurally diverse environmental pollutants including pesticides, toxic industrial waste, direct and second hand cigarette smoke, vehicle exhaust, urban air pollutants, ozone, radiation, and physical stress contribute to the excessive generation of free radicals, which in turn pose significant risks to human health [48].

Oxidative stress has been implicated in several pathological processes, including mutagenesis, carcinogenesis, lipid peroxidation, disruption of membrane integrity, and protein oxidation and fragmentation. Therefore, maintaining a proper balance between prooxidant and antioxidant compounds within the body is considered a key strategy in the prevention of cardiovascular, oncological, and neurodegenerative diseases [49]. 

In vitro, the phytochemicals found in *Ut* and green tea decoctions appear to be more effective antioxidants than vitamin C in some cell lines and at concentrations that suggest that they may be acting at distinct levels, inherent to their ability to interact with the injurious oxidant, suggesting that they act on intracellular pathways regulating cell death [50]. These data indicate that dietary supplementation with these or related antioxidants may prove to be valuable in limiting the pathophysiology of numerous disorders associated with gut inflammation [50].

A research work with aqueous and ethanolic extracts from bark and leaves of *Ut* demonstrated their protective activity on human erythrocytes in oxidative stress induced by 2,4-dichlorophenol. These activities were attributed to the presence of polyphenols, especially hydroxicinnamic acids, flavonols and polymeric proanthocyanidins [51]. Their antioxidant properties were demonstrated by the significant reduction of various free radicals and oxidative species, and by the protection of the sarcoplasmic reticulum membranes from lipid peroxidation, activities that were related to proanthocyanidins [4,52]. In 2018, Navarro-Hoyos et al. revealed these ethanolic extracts as being potentially marketable, being rich in proanthocyanidins and exhibiting high antioxidant activity [53].

Effects of aqueous and ethanolic extracts derived from the leaves and bark of *Ut* were also investigated in terms of the osmotic resistance of human erythrocytes, intracellular viscosity, and plasma membrane fluidity. All tested extracts led to a reduction in membrane fluidity and an increase in erythrocyte osmotic sensitivity. Notably, the ethanolic extracts from both bark and leaves also elevated intracellular viscosity, the most pronounced changes across all evaluated parameters being observed in erythrocytes treated with the ethanolic bark extract. The authors found that the ethanolic extracts were characterized by a higher alkaloid content than the aqueous extracts, being richer in polyphenols [54].

Recently, a hydroalcoholic extract from *Ut* leaves was found to contain the highest concentration of quinovic acid derivatives and exhibited potent free radical-scavenging activity, with IC_50_ values of 0.113 μg/mL in the DPPH assay and 9.51 μM in the FRAP assay. The extract also inhibited superoxide anion generation by 77.3 ± 0.69% and suppressed nitric oxide (NO) production by 47.1 ± 0.37% at a concentration of 40 μg/mL. Furthermore, molecular docking studies indicated a potential interaction between quinovic acid and CLASP proteins, suggesting possible implications for the treatment of neurodegenerative diseases [54].

### 4.2. Anti-Inflammatory Activity

Inflammation is a multifactorial cellular response that plays a fundamental role in maintaining homeostasis when the organism is challenged by harmful agents or mechanical tissue injury. This response is characterized by a marked increase in the infiltration of polymorphonuclear leukocytes and monocytes into the affected tissue, accompanied by the release of various inflammatory mediators. Tumor necrosis factor-alpha (TNF-α) is a potent proinflammatory cytokine and a key mediator of chronic inflammatory conditions [29]. TNF-α exhibits cytotoxic effects on lymphocytes and, under certain circumstances, may contribute to immunosuppression. Nonetheless, TNF-α and other proinflammatory cytokines including interleukin-1 (IL-1), interleukin-6 (IL-6), chemokines, growth factors, and enzymes such as cyclooxygenase-2 (COX-2) and inducible nitric oxide synthase (iNOS) are typically encoded by quiescent genes under physiological conditions.

However, inflammation, immune activation, and infection trigger the activation of several transcription factors, ultimately leading to changes in gene transcription and translation. Among these, nuclear factor kappa B (NF-κB) plays a pivotal role in regulating nuclear events that promote cell survival and drive the production of proinflammatory cytokines. NF-κB modulates the expression of several cytokines, including tumor necrosis factor-alpha (TNF-α); interleukins IL-1, IL-2, IL-6, and IL-8; as well as mediators of the inflammatory response such as lipopolysaccharide (LPS) and mitogen-activated protein (MAP) kinases [55,56,57]. Inhibition of NF-κB has been associated with anti-inflammatory and antimutagenic effects, as it reduces the production of proinflammatory cytokines and thereby limits the generation of endogenous free radicals. This inhibition helps to prevent excessive or prolonged inflammatory responses, which can be harmful to surrounding healthy tissues and may result in cumulative genetic damage if not adequately resolved by the DNA repair mechanism [20]. Below optimal conditions, inflammation leads to the complete restoration of tissue integrity. However, when this process is not properly regulated, it may cause collateral damage to cellular and extracellular components adjacent to the inflamed site, resulting in the development of chronic inflammatory disease.

Crohn’s disease and ulcerative colitis are the two most prevalent forms of inflammatory bowel disease (IBD). Although the exact aetiology of IBD remains unclear, growing evidence implicates reactive oxygen species, free radicals, and intestinal microbiota in its pathogenesis [12]. These oxidative and inflammatory processes are also closely associated with a range of other chronic conditions, including atherosclerosis, rheumatoid arthritis, and various cancers. Notably, it is estimated that inflammation-related mechanisms contribute to the pathogenesis of over 30% of human malignancies.

*Ut* is globally recognized and widely utilized, primarily for its anti-inflammatory effects, a fact that has been consistently demonstrated across numerous studies employing various types of extracts. In an in vivo study, the activity of two bark extracts were compared, an 80% aqueous ethanol extract and a commercial aqueous freeze-dried extract. They contained 5.61% and 0.26% of oxindole alkaloids, respectively; the hydroalcoholic extract showed higher indomethacin activity than the aqueous extract and had a greater inhibitory effect on the activity of NF-κB. However, the aqueous extract was more effective than the hydroalcoholic one in inhibiting cyclooxygenases. Pentacyclic oxindole alkaloids, present in the extracts, are suggested as the bioactive compounds; these OAs are known to modulate the cellular immune system by affecting T and B-lymphocytes, influencing phagocytosis and inhibiting the activation of lymphocyte proliferation [58].

According to Sandoval-Chacón’s American research team, the administration of 100 µg/mL of *Ut* extract containing a mixture of quinovic acid and glycosides as well as pentacyclic or tetracyclic of oxindole alkaloids such as pteropodine, isopteropodine, speciophylline, uncarine F, mitraphylline and isomitraphylline, attenuated peroxynitrite-induced apoptosis. *Ut* also inhibited the expression of inducible genes associated with inflammation, so it prevents the activation of the transcription factor NF-κB. The anti-inflammatory properties were verified at doses that are consistent with the practice of traditional medicine, which provides strong evidence for the widely held belief that *Ut* is an effective anti-inflammatory agent [12].

In a study with a decoction from the bark of *Ut*, anti-TNF-α effects at doses as low as 1.2–30 ng/mL were observed. This is particularly impressive considering that an extract is not a pure phytochemical, which suggests that *Ut* bark may be an excellent adjunctive to therapy in chronic inflammation. The results of this study suggest that the antioxidant and anti-inflammatory properties of cat’s claw are independent of their oxindole or pentacyclic alkaloid content, suggesting that other compounds present in the plant are responsible for these effects [35].

Instead, treatment with *Ut* ethanolic extracts obtained by exhaustive percolation with 95% ethanol, containing mainly uncarine F, mitraphyllene, speciophylline and isopteropodine, was found to inhibit LPS-induced TNF-α secretion, while paradoxically, enhancing LPS-induced IL-1β production. This differential modulation of cytokine responses may hold clinical relevance, particularly considering that targeted inhibition of TNF-α, commonly achieved through antibody-based therapies, has been employed in the management of chronic inflammatory conditions such as rheumatoid arthritis and Crohn’s disease. Additionally, *Ut* extracts suppressed activation of the MAP kinase pathway in a dose-dependent manner, without promoting cell death. Despite these promising properties, the authors did not establish any relationship between the activities of the extracts and the compounds present [55].

Moreover, significantly lower levels of protein in bronchoalveolar lavage fluid, a lower degree of epithelial necrosis, a higher number of intact epithelial cell nuclei in the bronchial wall, and a decreased number of infiltrating neutrophils in the bronchial lumen were verified in male mice exposed to ozone (O_3_) after treatment with an *Ut* decoction. Ozone has been associated with respiratory tract inflammation and lung functional alterations, and *Ut* extract probably prevented ozone-induced respiratory inflammation in male mice. The authors did not perform a chemical analysis of the extract, thus precluding the identification of its bioactive compounds [59].

Some studies indicate that alkaloids seem not to interfere with anti-inflammatory activity [35], but it can result from a synergic interaction of different phytochemicals. In fact, an aqueous extract called C-Med-100, with low amounts of alkaloids (<0.05%), was shown to be very effective in the treatment of several health disorders, including chronic inflammation and gastrointestinal dysfunction [45]. Furthermore, inhibition of TNF-*α* and nitric oxide in LPS-induced macrophages was verified in multiple fractions containing non-alkaloid polar compounds [35]. Consequently, it is unlikely that a single substance could be responsible for all of these therapeutic activities.

More recently, Dietrich and his colleagues (2015) demonstrated the promising anti-inflammatory properties of the quinovic acid glycosides purified fraction from *Ut* against haemorrhagic cystitis induced by cyclophosphamide in mice [60], this effect having been previously reported by Figueiredo and his colleagues (2012) [61].

Considering that proanthocyanidins are metabolized by the action of gut microbiota, the results of Navarro-Hoyos his team (2017) show the potential health effects of *Ut* proanthocyanidin extracts on gut-related diseases, such as colon cancer, and according to these findings, it would be of interest to develop novel commercial dietary ingredients or botanical drugs derived from *Ut* [62].

Lately, Lima and coworkers (2020) highlighted the role of phytochemicals from *Ut*, as a safe anti-inflammatory, but also as an antiresorptive and a potential bone anabolic product, to be considered as a therapeutic alternative to control bone diseases, opening new avenues for a further clinical study [63].

### 4.3. Immunostimulation Activity

Substantial historical, empirical and scientific evidence have demonstrated that plants have immunomodulating activity [64]. Four oxindol alkaloids isolated from the roots of *Ut* (pteropodine (2), isopteropodine (5), isomitraphylline (6), and isorynchophylline (8)) enhanced the activity of phagocytes both in vitro and in vivo [65]. Other results suggested that there might be some additional active components other than alkaloids in hot water extracts of *Ut*, such as C-MED-100^TM^, that could contribute to immune enhancement [11]. In fact, a study in rats with C-MED-100^TM^ depleted of large molecular weight conjugates and of indole alkaloids evidenced a dose dependent enhancement of phytohemaglutinin (PHA), stimulating lymphocyte proliferation and increasing white blood cells [11]. Investigations with aqueous extracts of *Ut* stem bark from different areas of Peru showed their potent stimulant action on alveolar rat macrophages, increasing their production of interleukin-1 and interleukin-6 [66].

In another study with human volunteers, a 350 mg tablet of C-MED-100^TM^, a hot water extract free of oxindole alkaloids and high molecular weight conjugates, was provided twice a day for two months. A pneumococcal vaccine was administered to all individuals 30 days after beginning the supplementation with C-MED-100^TM^. No toxic side effects were observed, but a statistically significant immune enhancement, verified by an elevation in the lymphocyte/neutrophil ratio of peripheral blood and a reduced decay in pneumococcal antibody response, was observed after 5 months [67]. To gain further insight into the basis of this immunomodulatory effect, mice were supplemented with C-Med 100^TM^. A dose-dependent increase in spleen cell numbers was observed; however, the proportions of B cells, T cells, natural killer (NK) cells, granulocytes, and memory lymphocytes remained within normal ranges. Notably, no alterations were detected in the lymphoid architecture of the spleen, even after prolonged treatment. Upon discontinuation of supplementation, spleen cellularity returned to baseline levels within four weeks. These findings indicate that C-Med 100™ significantly prolongs lymphocyte survival in peripheral lymphoid organs without enhancing their proliferation rate, suggesting its potential utility as a therapeutic agent to accelerate immune recovery in patients with leukopenia [68].

To better characterize the stimulatory activity of *Ut* on the cellular constituents of the immune system, the hematopoietic response in mice infected with *Listeria monocytogenes* was evaluated after they were treated with an extract containing 1% of total alkaloids. This Gram-positive, facultative intracellular bacterium has been widely employed as an experimental model to investigate the mechanisms underlying innate and cell-mediated antimicrobial defence. It primarily replicates within macrophages and elicits a robust innate immune response involving coordinated interactions among neutrophils, macrophages, natural killer (NK) cells, and T lymphocytes. In the early stages of infection, the production and mobilization of phagocytes from hematopoietic progenitors in the bone marrow to infected tissues are critical for controlling bacterial proliferation. This process is largely dependent on specific growth factors known as colony-stimulating factors (CSFs). The effects of oral administration of *Ut* extract on hematopoietic function were evaluated in mice by measuring the growth and differentiation of bone marrow and spleen colony forming units of granulocyte macrophages (CFU-GMs), as well as serum levels of CSFs, IL-1, and IL-6. Administration of 50 and 100 mg/kg of the extract for seven consecutive days prior to a sublethal bacterial challenge prevented infection-induced myelosuppression and restored spleen CFU-GM counts. Notably, the 100 mg/kg dose significantly upregulated CSF production, mirroring its effect on CFU-GM recovery. These findings demonstrate a stimulatory effect of the extract on the hematopoietic response in both infected and uninfected mice, potentially contributing to the enhanced protection observed during lethal infection. Consistent with previous studies, increased levels of IL-1 and IL-6 were also detected in non-infected mice treated with 100 mg/kg of *Ut* extract. Both cytokines are crucial for early resistance to listeriosis, with IL-1 additionally synergizing with IL-12 to induce interferon-γ (IFN-γ), a key activator of macrophages. Collectively, these results suggest that *Ut* extract indirectly modulates immune responses by enhancing the hematopoietic reserve through increased cytokine production (CSFs, IL-1, and IL-6), potentially counteracting Listeria-induced immunosuppression. Despite these promising results, this work has a flaw in not presenting information about the type of extract and its composition, and therefore, the bioactive compounds cannot be identified [69].

Immune responses induced in hens after oral administration of bovine serum albumin in combination with a dry extract from the bark of *Ut* concluded that the extract was useful as an oral adjuvant or immunomodulatory [70]. Moreover, a hydroalcoholic extract obtained from *Ut* cortex, tested on dendritic cells and HLA-DR/CD86 molecules after lipopolysaccharides stimulus, demonstrated its involvement in adaptative immune response mechanisms [71].

The immunostimulant activity of *Ut* bark decoction and its tannin-rich fraction (TF) on human macrophages involved increasing the expression of interleukins IL-1*β*, IL-6, IL-8, IL-10, and IL-12; granulocyte-macrophage colony-stimulating factor (GM-CSF); chemokine ligands CCL2, CCL3, and CCL4; and tumor necrosis factor (TNF)-α, which supports the traditional use of the plant as immunostimulant and points to its tannins as immune-modulating compounds that could play a significant role in human disease prevention and treatment [72]. A hydroalcoholic extract from the stem bark was also able to modulate distinct patterns of immune system activation in a dose-dependent manner, as well as being effective in preventing the progression of immune-mediated diabetes in mice. Although the authors determined the chemical profile of the extract used, they did not mention which compounds were responsible for which activities [73].

In accordance with Perejón-Rubio and García-Gimenez (2022), the proanthocyanidins of *Ut* prevented the exit of SARS-CoV-2 particles and interfered with the coupling of the complex these particles form with angiotensin-converting enzyme 2 (ACE-2). In addition, they impeded the virus’s replication and maturation, reducing biochemical and molecular alterations in infected cells [74].

### 4.4. Cardiovascular Protective Activity

The tetracyclic uncaria alkaloids rhynchophylline (7) and isorhynchophylline (8) mainly act on the cardiovascular system [75], where reportedly exert hypotensive and vasodilatory effects. Although some results suggest that they may act via multiple Ca^2+^ pathways, the mechanism of action is unclear [76]. Pharmacological studies on the tetracyclic alkaloids hirsutine (12), hirsuteine (13), rhynchophylline (7), isorhynchophylline (8) and dihydrocorynantheine (11) present in the tetracyclic oxindole alkaloid (TOA) chemotype of *Uncaria tomentosa* showed in vivo a hypotensive effect in rats that may be partly due to the vasodilation effect induced by its *α*-adrenoceptor blocking action. Moreover, hirsutine also had antiarrhythmic effects on both aconitine-induced and ouabain-induced arrhythmias in mice and guinea pigs, respectively [77], and rhynchophylline (7) inhibited in vivo platelet aggregation [78]. Another study indicated that hirsutine (12) and dihydrocorynantheine (11) had direct effects on the action potential of cardiac muscle through inhibition of multiple ion channels, which may explain their negative chronotropic and antiarrhythmic activity [79].

Nevertheless, there are other compounds in *Ut* that may contribute to this activity. In fact, according to Gonçalves et al. (2005), *Ut* bark decoction is mainly composed of phenolic acids, essentially caffeic acid (26) and proanthocyanidins [4]. In the context of cardiovascular health, a particular group of flavonoids, namely the flavan-3-ols polymers, has recently received attention [80]. 

The results derived from one study by Kolodziejczyk-Czepas (2021) on thrombin suggest that *Ut* may be a source of natural substances with anti-coagulant and anti-thrombotic properties, even if the extracts are weak antiplatelet agents [81].

Oogaki et al. (2021) demonstrated that the hot water AC-11^®^ commercial extract of *Ut* has the potential to regulate blood pressure by controlling the balance of T cell population and inflammatory cytokine production both in non-pregnant and pregnant conditions. A significant shortcoming of the work is the absence of details regarding the extract type and its composition, which prevents the identification of the bioactive compounds [82].

### 4.5. Neuroprotective Activity

Morphine is an alkaloid that has been extensively used in human history to alleviate pain. The antinociceptive activity of *Ut* was verified in chemical and thermal models of nociception in mice using a fraction containing 95% oxindole alkaloids. It was demonstrated that systemic administration of *Ut* causes a dose-dependent inhibition of the nociceptive behavioural response in mice subjected to these stimuli. It was concluded that there was an involvement of serotonergic mechanisms in this activity. Furthermore, the present study showed that this fraction produces antinociception that appears to be mediated by an interaction with 5-hydroxytryptamine_2_ (5-HT_2_) receptors [83].

On the other hand, there are also works with rhynchophylline (7) and isorhynchophylline (8), the major tetracyclic oxindole alkaloid components of *Uncaria* species, namely *Ut*. These alkaloids act on the cardiovascular system and also the central nervous system with effects such as protection against cerebral ischemia and sedation. The action mechanisms include calcium channel blockade, potassium channel opening, and modulation of neurotransmitter transport and metabolism, among others [75]. Rhynchophylline (7) has a protective effect against the damage induced by dopamine in the NT_2_ neuron cell line [84]. The protective effects of rhynchophylline (7) and isorhynchophylline (8) were demonstrated on in vitro ischemia-induced neuronal damage in the hippocampus and it was suggested that neurotransmitter receptors, like N-methyl-D-aspartate (NMDA), muscarinic M_1_ and 5-hydroxytryptamine_2_, were involved [85]. Later, in another study, it was found that isorhynchophylline (8) and isocorynoxeine (10) supress dose-dependently 5-HT_2A_ receptor function in mice. However, the inhibitory effect of this receptor–mediated behavioural response was not verified with its stereoisomers rhynchophylline (7) and corynoxeine (9) [86].

A study by Guthrie et al. (2013) demonstrated the role of carboxyl alkyl esters on recovery of sensorineural functions following exposure to a damaging level of noise [87]. Cosentino and Torres (2008) and Shi et al. (2013) demonstrated a possible therapeutic intervention against Parkinson’s disease, explained by its neuroprotective antioxidant activity [88,89].

A study with C-Med-100^TM^, a water soluble extract from *Ut*, improved the cognition, memory and learning, and minimized DNA damage, in the brain of middle-aged rats, explained by its bioactive phytochemicals that can be beneficial in aging and brain health, as well as providing novel insights into the mechanisms of neuronal aging [90]. Xu et al. (2021) found that *Ut* can ameliorate cognitive deficits in STZ-induced Alzheimer’s disease rats. The underlying molecular mechanism involved suppression of tau hyperphosphorylation, and the antioxidant and anti-neuroinflammatory activities were achieved via modulating Akt (Ser473)/GSK3 beta (Ser9)-mediated Nrf2 activation [91]. These findings amply suggest that *Ut* is worthy of being developed clinically into a pharmacological treatment for Alzheimer’s disease effects.

### 4.6. Protective Activity Against Cancer

Natural products have historically played a pivotal role in drug discovery and development, particularly in the field of oncology. An analysis of new anticancer agents approved by the United States Food and Drug Administration (FDA) between 1981 and 2019 revealed that more than 64% were of natural origin or derived from natural products [92].

A study was designed for further exploration of the anti-proliferative potency of some of *Ut’s* phytochemicals. Various preparations with different quantitative and qualitative alkaloid content were evaluated on HL-60 promyeolocytic leukaemia cells. The highest activity of a mixture of pteropodine (2)/isomitraphylline (6) and isopteropodine (5) suggested that the extract activity is probably related not only to oxindole alkaloids, but also other phytochemicals. On the other hand, there is no full agreement with previous evaluations of oxindole alkaloid cytotoxicity [37,93,94,95,96]. In other work about the cytotoxicity and genotoxicity of oxindole alkaloids from *Ut*, different selectivity against human malignant cells was verified, although the correct identification of each chemotype seems to be important to better understand its antitumor potential [97]. Another work demonstrated the antitumoral action of mitraphylline (3), a pentacyclic oxindole alkaloid from the inner bark of *Ut*. A potent antiproliferative effect was demonstrated in two human brain cell lines, SKN-BE(2) neuroblastoma and malignant GAMG glioma, with an IC_50_ of 12.3 and 20 µM, respectively, and the glioma cell line was more sensitive to this alkaloid that the controls vincristine and cyclophosphamide [14]. Notably, a significant pro-apoptotic activity in human medullary thyroid carcinoma cells (MTC cells) was verified for the alkaloids isopteropodine and pteropodine, whereas the alkaloid-poor fraction inhibited cell proliferation but did not induce apoptosis [15].

In an in vivo carcinosarcoma model, the antitumoral and antioxidant effects of an hydroethanolic extract of *Ut* were explained as the ability of the extract to regulate redox processes that probably play a pivotal role in the antiproliferative effects of the plant [95]. DNA repair is important in maintaining both cell viability and genomic stability; a cell responds to DNA damage in one of three ways: (1) by tolerating the damage; (2) by repairing the damage; or (3) by undergoing apoptosis. Tumourigenesis results from unrepaired damage. It seems that *Ut* extracts and their fractions from bark exert a direct antiproliferative activity on the growth of MCF-7, the most extensively used breast cancer cell line [98]. In another study, three different cell lines: SAOS (human osteosarcoma cell line), HeLa (human cervical carcinoma cell line) and MCF-7 were used in cytotoxicity assays with *Ut* root bark. The extract was prepared according to the prescribed formulation in traditional medicine, lyophilized, and the residue resuspended in water (120 mg/mL and 240 mg/mL) or *n*-butanol (240 mg/mL). The activity appears to be dose-dependent, with the higher doses showing a cytostatic effect and the inhibitory effect being stronger on HeLa cells. On the other hand, an induction of apoptosis by the n-butanol soluble fraction, via the activation of caspase 3, was also observed [99]. The mode of apoptosis induction is not known; in order to elucidate its molecular mechanism, various phytochemicals were extracted in another study, using four solvents of different polarities (*n*-hexane, ethyl acetate, *n*-butanol and methanol). Trypan blue exclusion and flow cytometry were used to detect the anti-cancer effect on HL-60, a leukaemia cell line. It was found that the ethyl acetate extract was particularly able to induce apoptosis, but also the *n*-hexane and *n*-butanol extracts had a stronger anti-cancer effect when compared with the methanol extract. Ethyl acetate extract induced cell death by cell body shrinkage and chromatin condensation, which is characteristic of apoptosis. Further investigations about the molecular mechanism of apoptosis allowed the discovery that it happens through reactive oxygen species production, cytochrome C release and the induction of caspases activity [23].

One study using cell lines and the water soluble fraction of the *Ut* extract C-MED-100, depleted of large molecular weight conjugates, demonstrated a dose-dependent inhibition of cell proliferation in two human leukaemia cell lines and one lymphoma cell line, which appeared to be mediated through induction of apoptosis [11]. In another study, C-MED-100^TM^ was given orally for eight consecutive weeks. One group was daily supplemented with a 250 mg tablet and the other with a 350 mg tablet of this aqueous extract. In the supplemented groups, an enhancement of DNA repair (both DNA damage and lethal DNA damage to the cell induced by radiation) was observed, and no drug-related toxic effects were noted [30].

On the other hand, chemotherapeutic agents are often drugs in which the therapeutic dose is many times restricted by the nonselective toxic effects on normal tissues. Haematological toxicity is the most dangerous form of toxicity for most of the antineoplastic drugs used in clinical practice. Both rats and human volunteers supplemented with C-MED-100^TM^ were observed to have an increased tendency for elevation of white blood cells (WBC). When using C-MED-100^TM^ to treat leukopenia induced by chemotherapy in a rat model, all fractions of WBC were proportionally elevated, and these results were also confirmed by microscopic examination. The mechanism is not known but all the results seem to support a general immune enhancement [100]. The extract used in these studies contained only trace amounts of alkaloids such as pentacyclic oxindole alkaloids (<0.05%), and so, consequently, these compounds could not be the only active phytochemicals [30,100]. These researchers proposed the carboxy alkyl esters (CAEs), i.e., quinic acid analogues, as active ingredients since the extract contains more than 8% of CAEs [20,30]. Recent studies evidenced that the hydroalcoholic extract of *Ut* has a role for cancer patients as a complementary therapy minimizing the effects of chemotherapy [101,102,103,104,105].

Ursolic acid, a triterpene present in *Ut*, is a phytochemical with numerous pharmacological activities. Cancer treatment constitutes the most auspicious uses, namely for colorectal, prostate, breast and liver cancer, either alone or in association with conventional drugs [106]. Furthermore, different studies have identified the capacity of ursolic acid to cause cancer cell apoptosis, prevent angiogenesis and inhibit resistance to drugs [107].

Gurrola-Díaz et al. (2011) investigated the inhibitory mechanisms of the water-soluble extract prepared from the bark of *Ut* (8 h at 37 °C) on the Wnt signalling pathway; three cancer cell lines displaying different levels of aberrant Wnt signalling activity were transfected with Wnt signalling responsive Tcf reporter plasmids and treated with increasing concentrations of the extract. It was observed there was an inhibition of the cancer cells, demonstrating the pharmacological action of this extract [108].

Santos et al. (2016) concluded that the hydroalcoholic extract of *Ut* bark may be responsible for the reduction of adenosine levels in the extracellular medium, and additionally, demonstrated it is effective when combined with chemotherapy [109].

In a complementary way and in accordance with Almeida et al. (2017), this medicinal plant can also effectively contribute to improving the quality of life (reducing fatigue) and the recovery of people undergoing chemotherapeutical treatments [110]. Zari et al. (2021) recently reported that *Ut* possesses significant anti-cancer properties. The *Ut* bark extracts exhibited significant anticancer activity both in vitro and in vivo, as demonstrated by its effects on the growth and survival of B16-BL6 mouse melanoma cells. Treatment of B16-BL6 cell cultures with both ethanolic and phosphate-buffered saline (PBS) extracts of *Ut* bark resulted in up to an 80% reduction in cell proliferation and a marked increase in apoptosis compared to vehicle-treated controls. Notably, the ethanolic extract was substantially more effective than the aqueous extract (decoction) in inducing these effects [111].

Recent studies have shown that *Ut* phytochemicals can inhibit P2X7 receptor-mediated breast cancer invasion and are expected to be used clinically [112].

### 4.7. Antibacterial Activity

*Uncaria tomentosa* and other eight promising medicinal plants have been used traditionally by local inhabitants of Callería District, Peru, for treating conditions likely to be associated with microorganisms.

In the case of ethanolic extract obtained from the bark of *Ut*, its antibacterial activity against *Bacillus cereus*, *Bacillus subtilis*, *Enterococcus faecalis*, *Staphylococcus aureus*, *Staphylococcus epidermidis* and *Escherichia coli* was proven at concentrations of 16 mg/mL or below [113]. As *Staphylococcus aureus* is related to nosocomial infections and shows resistance to several antimicrobials, it was also suggested that the use of *Ut* against this microorganism as a very promising strategy. It was also effective in inhibiting the growth of *Enterobacteriaceae*, which is associated with severe periodontal diseases [17,18].

Nowakowska and coworkers (2010) studied the effect of a bark aqueous extract on *Pseudomonas aeruginosa* infection in mice and concluded the inhibitory effect of doses 10 and 20 mg/kg as statistically significant; however, the highest dose (100 mg/kg) did not induce important changes in the number of bacteria [114].

Tay et al. (2015) demonstrated the effectiveness of 2% *Ut* gel on denture stomatitis caused by *Candida albicans* [115]. Additionally, Herrera et al. (2016) showed the antibacterial effect of a 2% *Ut* gel against *Enterococcus faecalis* in infected root canal dentin [116]. Recently, Dioguardi et al. (2022) demonstrated that the bioactive compounds present in *Ut* could represent an interesting product to be used in the endodontic field, both in endocanal cements and as a gel [117].

At the same time, Blanck et al. (2022) suggested that *Ut* phytochemicals may have antibacterial potential individually or synergistically with established antibiotics against microbes, including *Borrelia burgdorferi*, the causative agent of Lyme disease [118].

### 4.8. Antiviral Activity

In an age of emerging new viral diseases, like acquired immunodeficiency syndrome (AIDS), natural antiviral and immunomodulating compounds could play a significant role in human disease. However, relatively few antiviral drugs are available, and those approved for use often have high side-effects profiles and exhibit the potential to cause rapid resistance among targeted viral strains.

*Ut* extracts are also considered to have antiviral effects. In HeLa cells, it was reported they exhibit activity against rhinovirus type 1B infection, an effect due to a quinovic acid glycoside. Moreover the procyanidins cinchonain Ia (27) and Ib (28) and the 3-flavanol (-)-epicatechin (23) isolated from the bark of *Ut*, as well as a quinovic acid glycoside, were considered responsible for both anti-inflammatory and antiviral activity [6,38].

The antiviral and immunomodulating activities of a *Ut* hydroalcoholic extract containing pentacyclic oxindole alkaloids in vitro on human monocytes infected with Dengue virus-2 displayed novel therapeutic properties in dengue fever and might be further investigated as a promising candidate for clinical application [119]. Although further investigations should be carried out before a new drug can be suggested, a study in vitro, using a *Ut* alkaloid fraction, reduced paracellular permeability of and IL-8 and NS1 antigen production by human microvascular endothelial cells infected with Dengue virus [120].

Antiherpetic activity of different preparations of *Ut* was evidenced and justified by the presence of polyphenols, or their synergic effect with oxindole alkaloids or quinovic acid glycosides, since both purified fractions did not present activity; inhibition was the main mechanism of antiviral activity [121].

A study by Júnior et al. (2018) demonstrated the importance of using ethanolic extract of dried sliced bark from *Ut* to protect shrimp against white spot syndrome virus [122].

In 2020, as part of global efforts to identify effective therapies and preventive strategies against COVID-19, the crystal structure of the main protease Mpro (also known as 3CLpro) of severe acute respiratory syndrome coronavirus 2 (SARS-CoV-2) in complex with a synthetic inhibitor was resolved. This protease represents a key druggable target in the viral replication cycle. A multilevel computational study was subsequently conducted to assess the potential antiviral activity of phytochemicals derived from *Ut*, focusing specifically on Mpro inhibition. Structural bioinformatics approaches identified three promising bioactive compounds—speciophylline (4), cadambine, and proanthocyanidin B2, with strong predicted binding affinities for 3CLpro, suggesting potential therapeutic relevance [16]. Complementarily, Yepes-Perez et al. (2021) demonstrated that a hydroalcoholic extract of *Ut* stem bark inhibited the release of infectious SARS-CoV-2 particles by and mitigated virus-induced cytopathic effects on Vero E6 cells, providing the first experimental evidence of the antiviral properties of this plant against SARS-CoV-2 [123].

More recently, Yepes-Pérez et al. observed that the phytochemicals of the medicinal plant, such as proanthocyanidin C1, quinovic acid glycosides (QAG-2), 3-isodihydrocadambine, uncarine F (1) and uncaric acid, had good predicted binding affinity for the interface RBD–angiotensin-converting enzyme-2 (ACE-2) complex as compared to the sulfated heparin octasaccharide. Likewise, 3-dihydrocadambine and proanthocyanidins B4, B2 and C1 had the highest docking score with SARS-CoV-2 spike glycoprotein in their open state, whereas molecular dynamics simulations at 50 ns demonstrated both the feasibility of the binding free energy predicted by docking protocols and the stability of the docked protein–ligand complexes. These findings suggest that an herbal supplement at both preclinical and clinical stages should be evaluated for its effectiveness in the treatment of novel coronavirus disease (COVID-19); furthermore, all phytochemicals found in *Ut* could work in synergism by different mechanisms to combat the spread of SARS-CoV-2 [124].

Concomitantly, Herrera-Calderon et al. (2022) concluded that the anthocyanidin-based compounds detected in the stem bark ethanol extract of *Ut* could be responsible for the potential antiviral activity against the SARS-CoV-2 Omicron variant, the proanthocyanidin-C1 being considered as a powerful tool to combat new variants [125].

### 4.9. Skin Protective Activity

The skin, the largest organ of the human body, exercises functions as the primary defensive barrier protecting internal tissues from environmental insults. In line with this protective role, the skin is capable of absorbing nutrients, metabolites, and toxins from both internal and external sources. As a result, it is often the first organ to exhibit visible signs of aging. Topical application of antioxidants has been shown to reduce DNA and other macromolecular damage, representing a promising strategy for skin protection and repair. In this context, the potential of aqueous extracts of *Ut* to enhance skin resilience against ultraviolet (UV) radiation was investigated using primary skin organ cultures. The results demonstrated that co-incubation with C-Med-100™, an aqueous *Ut* extract, significantly reduced UV-induced skin cell death. This protective effect was attributed to an associated increase in DNA repair activity. These findings support the conclusion that C-Med-100™, which contains approximately 8% carboxyalkyl esters (CAEs) as active constituents, may offer a natural approach to photoprotection by enhancing the skin’s endogenous repair mechanisms [20].

### 4.10. Oestrogenic Activity

Aqueous extracts obtained from *Ut* bark seem to interact with distinct binding sites on the oestrogen receptor [19], which may be related to its particular contraceptive action. In fact, a significant reduction of oestradiol-specific binding with the aqueous extract of *Ut* was detected following 1 h of exposure to this extract [19].

A study carried out by Nogueira Neto et al. (2011) investigating experimental endometriosis reduction and contraceptive effects in rats showed that an *Ut* commercial extract (100 mg) appears to be a promising alternative for treating endometriosis [126]. The main issue with these studies is that the authors do not characterize the extracts or indicate which compounds are responsible for these activities.

## 5. Pharmaceutical Preparations

Taking into account the important and varied pharmacological activities of *Ut* previously described, it is imperative to refer to the scientific research carried out on pharmaceutical preparations. Several formulations of *Ut* (e.g., tinctures, decoctions, capsules, extracts and teas) are available on the market; however, modern pharmaceutical dosage forms involving *Ut* and another plant extracts are much less reported.

The anticancer properties of *Ut* have been mainly attributed to the pentacyclic oxindole alkaloids and its anti-tumorigenic activity can be improved significantly with nanotechnology. In fact, tumour tissues have a neovasculature composed of an endothelium with large fenestrations (from 200 to 780 nm) that allow nanoparticle permeation and accumulation in these tissues. This characteristic allows site-specific targeting of active agents and, in doing so, helps to avoid side effects. Ribeiro et al. (2013) prepared nanoparticles of poly epsilon-caprolactone (PCL) loaded with *Ut* dried extract (4.5% of total alkaloids) by the oil-in-water emulsion solvent evaporation method [127]. In 2015, poly-d,l-lactide-co-glycolide (PLGA) nanoparticles were developed, containing the same *Ut* extract, by the previous process [128]. These drug-loaded nanostructures provide several advantages, including protection against degradation, enhancing drug solubility and bioavailability, and sustained delivery of the drug, and due to their nanometric size, improved targeting of the drug to specific sites within the body. This strategy increases the efficacy of the drug and minimizes side effects. Ribeiro et al. (2020) used PCL and PLGA to generate nanoparticles loaded with *Ut* dried extract (4.5% of total pentacyclic oxindole alkaloids) by a single emulsion solvent evaporation method [129]. The results confirmed that the incorporation of *Ut* into nanoparticles could enhance its anti-cancer activities, which can offer a viable alternative for the treatment of prostate cancer and highlights the potential of nanostructured systems to provide a promising methodology to enhance the activity of natural extracts.

In order to standardize a spray-dryer from *Ut* bark hydroalcoholic extract, microcapsules were prepared and characterized [130]. The microcapsules did not present chemical incompatibilities or difficulties in the release of phytochemicals and maintained the content of active phenolic compounds as polyphenols, flavonoids and catechin derivatives, as well as its antioxidant activity.

In another work by Araya-Sibaja et al. (2022), the bioactivities of proanthocyanidins from *Ut* leaves were reported. When administered via nanosystems, these proanthocyanidins enhanced the cellular response in mice, confirming their role in immune modulation [131].

Considering that fast dissolving oral films is a new popular strategy of drug delivery with improved patient compliance and that this drug delivery system has answered the problems of solubility, bioavailability, improved biological half- life and therapeutic efficacy, Sowjanya and Rao (2024) formulated this dosage form containing *Ut* bark extract and evaluated its effect using in vitro studies for osteoarthritis. The results suggest that the fast dissolving oral films of *Ut* extract is novel, attractive and an alternative to the available marketed products, resulting in improved patient adherence in the treatment of this illness [132].

## 6. Safety and Toxicity

Hundreds of natural products are available to the consumer, although some could be potentially toxic when ingested in overdoses or in combination with other medications. Phytomedicinal preparations of *Ut* have been used traditionally among the indigenous peoples of South America. They are widely available commercially in the United States of America and other industrialized countries as alternative medicines.

Studies about the potential toxic effects of aqueous and hydroalcoholic extracts from *Ut* barks, roots and leaves were performed, evaluating different concentrations in animals. The extracts were well tolerated and showed low or no toxicity at the concentrations tested [133]. In an in vivo study, ten mice were treated with root aqueous extract of *Ut* containing 35 mg of pentacyclic oxindole alkaloids per g, as a 40% suspension. At a maximum dosage volume of 40 mL/kg bodyweight, the death of two of ten mice occurred within 4 h of treatment. It was revealed they experienced haemorrhage of the stomach and intestines and a pallor of the liver and spleen. The acute median lethal dose (LD50) of the aqueous extract was found to be greater than 16 g/kg bodyweight [22]. Moreover, it was also demonstrated that *Ut* bark aqueous extract, applied in a form of certificated preparation to calves, had no toxic properties with regard to the routine haematological parameters of erythrocytes or platelets. Therefore, it was concluded that this extract is a safe medicine for the haematopoietic system and it can be useful as a moderate enhancer of cellular immunity in calves [134]. In a study with C-MED-100^TM^ using both animals and humans, the LD 50 and the Maximum Tolerable Doses (MTD) were observed to be greater than 8 g/kg, which makes it comparable to the LD 50 of table salt (8–10 g/kg).

In short, the available toxicological studies did not demonstrate severe toxicity associated with oral intake of *Ut* preparations, suggesting a low potential for both acute and subacute oral toxicity.

Additionally, a topical formulation of C-Med-100^TM^ at a concentration of 5 mg/mL showed no histopathological signs of cytotoxicity in human skin culture assays, irrespective of ultraviolet (UV) exposure. On the contrary, C-Med-100™ exhibited a protective effect against UVB-induced sunburn and cell death. Nevertheless, this study did not evaluate the potential toxic effects of systemic absorption of C-Med-100™, nor did it assess the cumulative impact of repeated UVB exposure [20]. In 2014, Aguinaga et al. (2014) demonstrated that an aqueous extract of *Ut* bark had low toxicity to the fish *Hyphessobrycon eques* [135].

Despite the beneficial effects reported by the users, the indiscriminate use of *Ut* extracts without any medical criteria can be dangerous in some cases; moreover, there are few studies about drug interactions and side effects of medicinal plants like *Ut*. It is known that it should not be used by patients with an organ transplant or skin grafts who are using immunosuppressant drugs due to its immunostimulant properties [1]. Radiobiocomplexes such as sodium pertechnetate (Na^99m^TcO_4_) are tracers widely used in scintigraphic studies (single-photon emission computed tomography) mainly of the thyroid, but also of the brain and stomach. A study in vivo of Wistar rats evaluated the effect of aqueous preparations of a commercial *Ut* extract (Cats Claw, Herbarium Foundation for Health and Research, Curiti-ba, PR, Brazil, Lot No. 830283) on the biodistribution of this radiobiocomplex. The results obtained suggest that *Ut* extract can act in a meaningful way on the biodistribution of Na^99m^TcO_4_, in specific organs, such as heart, pancreas and muscle. Although these results were obtained with animals, caution is advisable in the interpretation of nuclear medicine examination when the patient is using this plant because it could lead to misdiagnosis with unexpected consequences [136].

To note, applying the chicken embryo model brought new information about the biological activity of *Ut*, showing unfavourable effects on some morphological blood parameters [137]. In addition, when administered together with diazepam, this plant enhanced the action of this benzodiazepine on spontaneous motor activity and exploratory ability [138].

In summary, the findings reported in the literature indicate that *Ut* is not genotoxic, although it does exhibit cytotoxicity. This cytotoxicity has been observed in studies employing both aqueous and hydroalcoholic extracts, as well as in isolated phytochemicals such as alkaloids and glycosides. These effects have been evaluated across various biological models, including non-neoplastic and neoplastic human cells, *Drosophila melanogaster*, Wistar rat cells, murine cells, and zebrafish (*Danio rerio*). In addition to its potential antimutagenic and antigenotoxic properties, *Ut* demonstrates protective activity against DNA damage, possibly attributed to the antioxidant or synergistic effects of its bioactive compounds, particularly alkaloids and glycosides, which may act by neutralizing free radicals. Overall, the evidence supports the safe use of *Ut* with respect to its toxicity profile [97,110,139,140].

Despite the reported data, toxicological studies must proceed following the development of new dosage forms.

## 7. Conclusions

The data reported in this review presented the current state of knowledge about the chemical composition, pharmacological activities, safety and toxicity of *Uncaria tomentosa*. Phytochemical studies have shown the presence in *Ut* of a diverse range of bioactive metabolites: alkaloids, triterpenes, glycosides and phenolic compounds, but it is controversial as to which compounds can be attributed to which activities. Different studies indicate that the various activities can result from a combination of phytochemicals that might synergise. Pharmacological studies indicated that this plant and its phytochemicals possess various biological activities, especially antioxidant, anti-inflammatory, antiviral, antibacterial, immunostimulant, estrogenic, neuroprotective, skin protective, cardiovascular protective, and protective against cancer. Due to its therapeutic properties, crude drugs and a variety of extracts have been commercialized worldwide and modern dosage forms are about to emerge.

Further studies are needed to determine the potential of *Ut* commercial products. Purification of extracts and an evaluation of the qualitative and quantitative phytochemical profiles of *Ut* extracts, and structure–bioactivity relationships with regard to different bioactivities and toxicity, especially for animals and humans, are very important to ensure its efficacy and safety and would be of interest to develop phytopharmaceutical drugs derived from *Ut*.

The exploration of novel phytopharmaceuticals derived from commonly utilized plant raw materials in traditional medicine holds the potential to facilitate the establishment of more readily accessible therapeutic alternatives for the general public.

In conclusion, this present review provides information on the ethnopharmacology, phytochemistry and pharmacology of *Ut*, which supports its further clinical use in modern medicine.

## Figures and Tables

**Table 1:** Chemical nature and distribution of alkaloids from *Uncaria tomentosa* [22,31].

	Pentacyclic Alkaloids	Tetracyclic Alkaloids
Oxindole alkaloids	uncarine F (1) L, R, S pteropodine (2) L, R, S mitraphylline (3) L, R, S speciophylline (4) L, R, S isopteropodine (5) L, R, S isomitraphylline (6) L, R, S	rhyncophylline (7) R, L, S isorhyncophylline (8) R, L, S corynoxeine (9) R, L isocorynoxeine (10) R, L
Indole alkaloids	akuammigine (15) L, R tetrahydroalstonine (16) L isoajmalicine (17) L	dihydrocorynantheine (11) R, L, S hirsutine (12) R, L, S hirsuteine (13) R, L, S corynantheine (14) R, L

L—leaves; R—roots; S—stems.
